# A reference to assess cilium phenotype in ciliopathy patients

**DOI:** 10.1186/2046-2530-4-S1-P60

**Published:** 2015-07-13

**Authors:** M Oud, H Arts, R Roepman

**Affiliations:** 1 Radboud Institute for Molecular Life Sciences Radboud University Medical Centre, Nijmegen, The Netherlands; 2 Department of Human Genetics, Radboud University Medical Centre, Nijmegen, The Netherlands; 3 Robarts Research Institute, University of Western Ontario, London, Ontario, Canada; 4 Department of Biochemistry, University of Western Ontario, London, Ontario, Canada

## Objective

The cilium presence and constitution of human fibroblasts are accessible and measurable cellular markers for ciliary homeostasis. Fibroblasts are most commonly generated from skin biopsies and comparison of the ciliary phenotype of patients vs controls can be used to identify or evaluate a potential ciliary defect. Subtle differences are difficult to detect, but critical to establish a genotype-phenotype correlation. The aim of this study is to establish the parameters to detect subtle differences in cilium phenotype, and develop a reference for cilium phenotype that can be used to improve diagnosis and prognosis of ciliopathies.

## Methods

We derived fibroblast cell lines from skin biopsies from ciliopathy patients and healthy controls. Ciliogenesis was induced by adding culture medium containing 0.2% FCS prior to immunocytochemistry. The cells were stained with antibodies against different ciliary proteins to study cilium presence, length, morphology, and intraflagellar transport proteins.

## Results

Fibroblasts from three controls and five patients with different ciliopathies were studied so far. We found major and subtle differences in cilium frequency and phenotype in patients and subtle differences in controls, partly influenced by the culturing conditions. This suggests that there is a significant natural and experimental variation that needs to be taken into account when evaluating the statistical relevance of such findings.

## Conclusion

We detected major and subtle differences in cilium phenotype between patients. In addition, we found a natural variation in the cilium phenotype of control fibroblasts. By including more samples, we intend to improve and expand this reference for ciliopathy-associated cilium phenotypes.

